# Diagnostic Comparison of Ultrasound and Magnetic Resonance Imaging in Detecting Rotator Cuff Tears: A Study Conducted in the Population of Vijayapura

**DOI:** 10.7759/cureus.68302

**Published:** 2024-08-31

**Authors:** Jayanth Ganesh, Satish D Patil, Rajashekar Muchchandi, Sandeep Naik

**Affiliations:** 1 Department of Radiology, Shri B. M. Patil Medical College, Hospital and Research Center, Bijapur Lingayat Education Association (Deemed to be University), Vijayapura, IND; 2 Department of Orthopedics, Shri B. M. Patil Medical College, Hospital and Research Center, Bijapur Lingayat Education Association (Deemed to be University), Vijayapura, IND

**Keywords:** shoulder pain, partial-thickness and full-thickness tears, rotator cuff tears, mri, ultrasound

## Abstract

Background: Injuries to the shoulder and restricted range of motion often lead to decreased work productivity, increased use of medical resources, and impaired quality of life. The most frequent cause of shoulder discomfort and dysfunction is a disease related to the rotator cuff, such as bursitis, degenerative tears, and calcific tendinosis. This study evaluates ultrasonography's diagnostic efficacy in relation to magnetic resonance imaging (MRI).

Methodology: Prospective research was conducted at a hospital to compare MRI and ultrasonography for shoulder cases involving rotator cuff injuries. There were 53 patients in the sample. Those who presented with pain and dysfunction in the shoulder were given both an MRI and an ultrasound (USG). Comparing the results of the MRI and USG allowed for the calculation of the diagnostic tests' accuracy, sensitivity, specificity, and positive and negative predictive values.

Results: The results of the study demonstrated a substantial agreement (p value <0.05) between the identification of rotator cuff tears by MRI and USG shoulder imaging. With a total accuracy of 88.6%, the sensitivity and specificity of identifying rotator cuff tears were 91.2% and 81.8%, respectively.

Conclusion: With similar sensitivity and specificity, MRI and USG are useful diagnostic techniques for rotator cuff injuries. USG is a great screening alternative due to its cost-effectiveness, noninvasiveness, and easy accessibility. However, when it comes to identifying the anatomical regions that need surgical repair, MRI is superior.

## Introduction

The human shoulder joint is a complex and well-balanced anatomic system capable of applying force in various directions and orientations. The glenohumeral joint (GHJ) primarily facilitates movement in the shoulder, whereas the scapulothoracic articulation also contributes significantly. The upper limb and scapula are connected through the acromioclavicular and sternoclavicular joints. The shoulder allows for a wide range of motion because of the shallow cup produced by the glenoid and the comparatively big humeral head. However, this design is fundamentally unsteady, akin to the challenge of balancing a golf ball on a tee. A multitude of static and dynamic structures supports the stability of the GHJ. The scapular, deltoid, and rotator cuff muscles are examples of dynamic structures. The glenoid labrum and related capsuloligamentous elements are among the static structures [1].

The humeral head is supported by the four muscles that make up the rotator cuff. Their tendons originate from the front and rear portions of the scapula and are inserted into the greater and lesser tuberosities. The subscapularis, which supports the shoulder anteriorly, the supraspinatus, which supports the shoulder superiorly, the infraspinatus, and the teres minor, which supports the shoulder posteriorly, are the muscles that make up the rotator cuff [2]. Rotator cuff disease, such as bursitis, degenerative tears, and calcific tendinosis, is the most prevalent cause of shoulder discomfort and dysfunction [3]. Shoulder pain and limited range of motion are among the most common causes of impaired quality of life, increased use of medical resources, and decreased workplace productivity [4].

Changes in tendinopathy leading to partial- to full-thickness tears primarily affecting the supraspinatus tendon and potentially involving the infraspinatus and/or subscapularis tendon [5] cause rotator cuff failure. Rotator cuff failure occurs most commonly after the age of 40, and in young people, it is uncommon and mainly occurs in athletes due to acute injuries [6].

The muscle fibers of the supraspinatus, infraspinatus, teres minor, and subscapularis become damaged or detached, resulting in a rotator cuff tear. The muscle most often affected is the supraspinatus. There are two forms of rotator cuff tears: partial-thickness tears, which involve only partial disruption, and full-thickness injuries [7]. External impingement, trauma, degenerative changes, lifting large weights, and joint instability can all result in tears [2].

Patients who have tears in their rotator cuffs often complain of shoulder pain and limited mobility. The most frequent underlying cause of shoulder pain, affecting between 65% and 70% of patients, is rotator cuff tears [8]. While a variety of imaging techniques have been employed to evaluate shoulder discomfort, the main imaging modalities for detecting rotator cuff injuries include ultrasound (USG), indirect and direct magnetic resonance arthrography (MRA), and unenhanced magnetic resonance imaging (MRI) [9].

Shoulder ultrasonography is being utilized increasingly often in hospital settings to assess the integrity of the rotator cuff. It is a noninvasive technique with no harmful effects that has the benefit of dynamically examining the tendons while the shoulder moves [10]. One drawback of the ultrasonography shoulder is that it requires a lot of operator experience and has a lengthy learning curve [11,12], particularly when partial-thickness rips are involved. It is not as good at examining the labrum, measuring the rotator cuff interval, or identifying small bone abnormalities. The sensitivity of shoulder ultrasonography is reduced in patients with significant movement restrictions or obesity [13].

Despite being a static modality, plain MRI is preferred because it may give high-resolution imaging in several planes. MRA can enhance its diagnostic capabilities by emphasizing shoulder abnormalities and aiding in differentiating intra-articular structures with an intra-articular injection of a radiopaque dye. Although MRA is a more effective diagnostic tool for shoulder conditions than traditional MRI, especially regarding rotator cuff integrity, the disadvantages of this invasive process must be considered in relation to the possible advantages. A radiology professional with extensive training is also necessary for MRI. The drawbacks of MRI encompass its accessibility, duration, cost, and strict prohibitions for patients with implanted infusion devices, cardiac pacemakers, biostimulators, metallic orbital foreign bodies, cochlear implants, automated defibrillators, and intracerebral aneurysm clips [9-12]. As a result, the primary goal of this study was to evaluate the diagnostic efficacy of ultrasonography relative to MRI.

## Materials and methods

Study design and setting

The Shri B. M. Patil Medical College, Hospital and Research Center, Vijayapura, hosted this hospital-based prospective research from September 2022 to June 2024. The study concentrated on patients who presented with pain and functional impairment to the Department of Orthopedics, especially clinical signs suggestive of rotator cuff injuries. These patients were evaluated for their shoulders at Bijapur Lingayat Education Association Shri B. M. Patil Medical College, Hospital and Research Center using ultrasonography and MRI technology. The institutional ethics committee gave its permission before the study could start.

Selection Criteria

Patients who met the inclusion criteria were those who presented with clinical symptoms suggestive of rotator cuff injuries and were then referred for shoulder MRIs and USGs. To qualify for inclusion, patients also had to be older than 18. Patients who were not eligible for MRIs, such as those with metallic implants or those who were claustrophobic, were excluded from consideration. Patients with known or diagnosed shoulder fractures or dislocations evident on plain radiography were also excluded from the study. Furthermore, individuals who had previously undergone shoulder surgery for any reason related to the rotator cuff were not considered eligible for participation.

Data sources and variables

The data collection method adhered to a systematic approach to ensure thorough assessment and accurate diagnosis. Before participating, informed written consent was obtained from all individuals involved. Each patient underwent a comprehensive review of their medical history and a brief physical examination conducted by board-certified orthopedic specialists with over 10 years of experience in diagnosing and managing shoulder pathologies. Following that, imaging tests were carried out, including shoulder MRIs and USGs, to identify and evaluate the severity of rotator cuff tears and associated disorders. The USG examinations were performed by a certified radiologist specializing in musculoskeletal imaging with more than eight years of experience. A GE Voluson S8 USG machine (GE HealthCare, Bengaluru), equipped with a linear-array transducer operating at 9 MHz, was used for the USG tests. The GE Signa Explorer (Medinnova Systems Private Limited, Vadodara), operating at 1.5 T, was employed for MRI scans of the shoulder.

The USG procedure followed a standardized protocol to achieve consistent and precise imaging of shoulder pathologies. Patients were positioned ideally in a sitting position on a revolving stool or the edge of a bed, ensuring optimal arm positioning and patient comfort during the examination. In cases where sitting was not feasible, a modified supine position was adopted, with the affected shoulder placed on the edge of the bed to facilitate imaging. This approach facilitated a comprehensive evaluation of shoulder anatomy and pathology while minimizing patient discomfort. For MRI shoulder scans, patients were positioned supine to facilitate imaging. A surface body coil was utilized for optimal signal reception and imaging resolution. This setup ensured effective visualization of shoulder anatomy and pathology, providing detailed diagnostic information for evaluating rotator cuff tears and related conditions.

Statistical analysis

After the data were imported into a Microsoft Excel sheet (Microsoft Corporation, Redmond, WA), the statistical analyses were carried out using the Statistical Package for the Social Sciences version 20 (IBM Corp., Armonk, NY). The results were presented using graphs, counts, percentages, averages, and standard deviations. The chi-square test was utilized to compare categorical variables between the groups. For all analyses that used two-tailed tests, a significance level of p < 0.05 was deemed statistically significant. Each patient's USG results were compared to their MRI results. The "within MRI" term specifies that the analysis considers how the USG results correlate with MRI outcomes for the same patient rather than comparing to an external standard or other diagnostic methods.

## Results

Table 1 summarizes the characteristics of 53 patients diagnosed with shoulder disorders. The mean age of the patients was approximately 48.6 years, with a median age of 50 years. In terms of age distribution, one patient (1.9%) was under 20 years old, four patients (7.5%) were between 20 and 29 years old, five patients (9.4%) were between 30 and 39 years old, 15 patients (28.3%) were between 40 and 49 years old, 17 patients (32.1%) were between 50 and 59 years old, and 11 patients (20.8%) were over 60 years old. The differences between age groups were statistically significant (p = 0.00025). Regarding gender distribution, 35 (66%) were male, and 18 (34%) were female. The difference between genders was statistically significant (p = 0.0195). The side affected was predominantly the right shoulder in 38 patients (71.7%), with the left shoulder affected in 15 patients (28.3%). The difference between the affected sides was statistically significant (p = 0.0016). The primary complaints reported were shoulder pain in 52 patients (98%), restriction of movements in 42 patients (79%), and trauma in 20 patients (37%).

**Table 1:** Characteristics of patients with shoulder disorders

Category	Subcategory	Number of patients (n)	Percentage (%)	Statistical significance (p value)
Age (years)	<20	1	1.9%	0.00025
	20-29	4	7.5%	
	30-39	5	9.4%	
	40-49	15	28.3%	
	50-59	17	32.1%	
	60+	11	20.8%	
	Total	53	100%	
Gender	Male	35	66%	0.0195
	Female	18	34%	
	Total	53	100%	
Side	Right	38	71.7%	0.0016
	Left	15	28.3%	
	Total	53	100%	
Complaints	Shoulder pain	52	98%	-
	Restriction of movements	42	79%	
	Trauma	20	37%	

Table 2 compares tear types detected by USG in 53 patients, revealing a strong correlation with MRI findings across various tear types. For rotator cuff tears, USG identified no tear in 13 patients (81.3% within MRI) and detected a tear in 34 patients (91.9% within MRI), with a chi-square test significance value of p = 0.001. Similarly, USG detected no supraspinatus tear in 17 patients (85.0% within MRI) and a tear in 30 patients (90.9% within MRI), with the same significance value. In the case of supraspinatus partial tears, USG identified no tear in 24 patients (88.9% within MRI) and a tear in 23 patients (88.5% within MRI), supported by a significance value of p = 0.0012. For full-thickness supraspinatus tears, USG found no tear in 43 patients (100.0% within MRI) and detected a tear in nine patients (90.0% within MRI), with a significance value of p = 0.0021. USG was also effective in identifying other tears (infraspinatus, teres minor, and subscapularis), detecting no tear in 36 patients (85.7% within MRI) and a tear in nine patients (81.8% within MRI), with a significance value of p = 0.004. These results highlight the high degree of alignment between USG and MRI in detecting various tear types, underscoring USG's accuracy and consistency as a diagnostic tool in this patient cohort.

**Table 2:** Comparison of tear types detected by USG No tear: patients in whom USG did not detect a tear, while the MRI findings for these patients confirmed that there was no tear Tear: patients in whom USG detected a tear, which was confirmed by MRI Within MRI: analysis considers how the USG results correlate with MRI outcomes for the same patient Total: the total number of patients assessed for each type of tear, which is consistent across all categories (53 patients) p value of <0.05 is considered to be statistically significant MRI: magnetic resonance imaging; USG: ultrasound

Tear type	Modality	No tear (count)	% within MRI	Tear (count)	% within MRI	Total	Chi-square test	Significance value
Rotator cuff tear	USG	13	81.3%	34	91.9%	53	28.354	p = 0.001
Supraspinatus tear	USG	17	85.0%	30	90.9%	53	30.540	p = 0.001
Supraspinatus partial tear	USG	24	88.9%	23	88.5%	53	31.710	p = 0.0012
Supraspinatus full thickness	USG	43	100.0%	9	90.0%	53	46.616	p = 0.0021
Others (infraspinatus, teres minor, and subscapularis)	USG	36	85.7%	9	81.8%	53	19.592	p = 0.004

Table 3 describes the diagnostic performance of USG in detecting various types of shoulder tears. The sensitivity of USG for detecting overall rotator cuff tears was 91.89%, with a specificity of 81.25%. For supraspinatus tears, the sensitivity was 90.91%, and the specificity was 85.00%. USG achieved a sensitivity and specificity of 100.00% for full-thickness supraspinatus tears, indicating perfect accuracy in this category. For partial-thickness supraspinatus tears, the sensitivity was 88.46%, with a specificity of 88.89%. When assessing other tears, including subscapularis, infraspinatus, and teres minor, the sensitivity was 81.82%, and the specificity was 85.71%. The positive predictive value (PPV) for overall rotator cuff tears was 91.89%, with a negative predictive value (NPV) of 81.25%. The PPV and NPV were 90.91% and 85.00% for supraspinatus tears, respectively. Full-thickness supraspinatus tears had a PPV and NPV of 100.00%, reflecting perfect diagnostic accuracy. The PPV and NPV were 88.46% and 88.89% for partial-thickness supraspinatus tears, respectively. The PPV for other tears was 60.00%, with a higher NPV of 94.74%. Finally, the overall accuracy of USG in diagnosing rotator cuff tears was 88.68%, with similar accuracy for supraspinatus tears and partial-thickness tears at 88.68%. Full-thickness supraspinatus tears had an accuracy of 100.00%, whereas the accuracy for detecting other tear types was 84.91%.

**Table 3:** Diagnostic performance of USG in detecting various shoulder tear types USG: ultrasound

Parameter	Overall rotator cuff tears	Supraspinatus tears	Full-thickness supraspinatus tears	Partial-thickness supraspinatus tears	Others (subscapularis, infraspinatus, and teres minor)
Sensitivity (%)	91.89	90.91	100.00	88.46	81.82
Specificity (%)	81.25	85.00	100.00	88.89	85.71
Positive predictive value (%)	91.89	90.91	100.00	88.46	60.00
Negative predictive value (%)	81.25	85.00	100.00	88.89	94.74
Accuracy (%)	88.68	88.68	100.00	88.68	84.91

## Discussion

Several methods, such as a clinical examination, X-ray, arthrography, USG, CT scan, and MRI, are used to assess individuals who express shoulder pain. While MRI is highly specific and sensitive, it is not always the primary imaging choice due to its high cost, limited availability, and contraindications in some patients. USG, being cost-effective, widely accessible, and capable of real-time dynamic assessment, is often preferred as a first-line tool for diagnosing rotator cuff tears, particularly in resource-limited settings [11]. The present study compared participants with clinical suspicion of rotator cuff tear and their USG and MRI findings.

Since each imaging modality has evolved over the years, it remains challenging to determine the best choice for evaluating rotator cuff tears, as numerous studies have compared the costs and effectiveness of USG versus MRI. The diagnostic effectiveness of USG has been significantly enhanced with the advent of high-frequency transducers and high-resolution sonography equipment. Similarly, the diagnostic quality of MRI has improved with the development of advanced MRI machines and better surface coils [14].

In the present study, the prevalence of rotator cuff tears was more common in males (66% of cases, n = 35) involving the right shoulder (72% of cases, n=38). The complaints given by the patient were mostly shoulder pain in 98% of cases (n = 52), and a history of trauma was present in only 37% of cases (n = 20). In the present study, the rotator cuff tear showed a significant correlation between USG and MRI, with a chi-square value of 28.1 and a p value of <0.05. The sensitivity and specificity for diagnosing rotator cuff tears were 91.89% and 81.25%, respectively, resulting in an overall accuracy of 88.68%. These results align with the findings of Teefey et al. [14], who reported that USG demonstrated a high sensitivity of 92% and specificity of 85% compared to MRI in detecting rotator cuff tears.

The supraspinatus muscle was the most commonly involved tendon in this study, with a prevalence of 62.6%. The correlation between USG and MRI for detecting supraspinatus tears had a chi-square value of 30.5 with a p value of <0.05. The sensitivity and specificity for detecting supraspinatus tears were 90.91% and 85%, respectively, with an accuracy of 88.68%. When compared to the study by McGuire et al. [15], which found USG to have an overall sensitivity of 88.3% for detecting supraspinatus tears, with a sensitivity of 83% for partial-thickness tears and 95.2% for full-thickness tears, the current study demonstrated slightly higher sensitivity for both partial- and full-thickness tears.

The partial-thickness supraspinatus tears in the current study showed a sensitivity of 88.46% and specificity of 88.89%, with an overall diagnostic accuracy of 88.68%. Full-thickness tears were detected with 100% sensitivity, specificity, and accuracy, consistent with the findings of Farooqi et al. [16], who reported high diagnostic accuracy of USG for full-thickness tears in their meta-analysis. For other tendons, such as the subscapularis, infraspinatus, and teres minor, the present study found a good correlation between USG and MRI, with a chi-square value of 19.5 and a p value of <0.05. The sensitivity and specificity were 81.82% and 85.71%, respectively, with an overall accuracy of 84.91%. These findings agree with the results of Okoroha et al. [17], who reported similar diagnostic accuracy of USG for these muscles. As USG is an inexpensive, noninvasive, and dynamic method with high sensitivity, accuracy values, and good correlation with MRI shoulder, it can be said that USG can be used as an initial investigating and screening tool for detecting rotator cuff tears in third-tier cities like Vijayapura.

Limitations of the study

A limitation of this study is the use of a 9-MHz linear probe for USG scans. While this probe is effective for detecting shoulder tears, the use of a higher resolution frequency probe, such as a 15 MHz probe, could potentially increase the accuracy of detecting smaller or partial-thickness tears, especially in muscles with lower tear prevalence like the subscapularis, infraspinatus, and teres minor. Additionally, despite the radiologist's extensive experience in musculoskeletal imaging, the inherent learning curve associated with shoulder USG may influence the detection rates of less common tear patterns.

## Conclusions

With comparable sensitivity and specificity, MRI and USG are useful diagnostic techniques for identifying rotator cuff injuries. Because it is widely available, noninvasive, and more cost-effective, USG is a great first screening technique for these injuries. Its real-time imaging capability allows for dynamic assessments, which can be beneficial in evaluating shoulder impingements. On the other hand, MRI provides superior detail in defining anatomical structures and is particularly useful for presurgical planning, helping to identify the precise locations where surgical correction may be needed. This makes MRI the preferred method when detailed anatomical information is required for treatment planning. Together, both modalities play complementary roles in the comprehensive evaluation of rotator cuff injuries.

## References

[1] Curl L, Warren R (1996). Glenohumeral joint stability. Selective cutting studies on the static capsular restraints. Clin Orthop Relat.

[2] Saraya S, El Bakry R (2016). Ultrasound: can it replace MRI in the evaluation of the rotator cuff tears?. Egypt J Radiol Nucl Med.

[3] Micheroli R, Kyburz D, Ciurea A, Dubs B, Toniolo M, Bisig SP, Tamborrini G (2015). Correlation of findings in clinical and high resolution ultrasonography examinations of the painful shoulder. J Ultrason.

[4] Takura T, Ushida T, Kanchiku T, Ebata N, Fujii K, DiBonaventura Md, Taguchi T (2015). The societal burden of chronic pain in Japan: an internet survey. J Orthop Sci.

[5] Lewis JS (2009). Rotator cuff tendinopathy. Br J Sports Med.

[6] Campbell RSD, Mistry A, Mc Nally E, Sellon E (2021). Internal derangements of joints: upper and lower limbs. Grainger and Allison’s Diagnostic Radiology.

[7] Craig R, Holt T, Rees JL (2017). Acute rotator cuff tears. BMJ.

[8] Anand A, Chhadi S, Bhawalkar S (2018). Comparison of ultrasound and MRI findings in rotator cuff injuries. Int J Contemp Med Surg Radiol.

[9] Roy JS, Braën C, Leblond J (2015). Diagnostic accuracy of ultrasonography, MRI and MR arthrography in the characterisation of rotator cuff disorders: a systematic review and meta-analysis. Br J Sports Med.

[10] Cox JL, Laughlin MS, Elkousy HA (2023). Determination of rotator cuff tear reparability: an ultrasound-based investigation. JSES Int.

[11] Griffith JF (2019). Top-ten pitfalls in rotator cuff ultrasound. Semin Musculoskelet Radiol.

[12] Barad HV, Patel V, Patel S, Patel M (2022). To determine the role of ultrasonography as a primary imaging modality as compared to MRI in patients with shoulder pain. J Family Med Prim Care.

[13] Selame L, Walsh L, Schwid M, Al Jalbout N, Gray MR, Dashti M, Shokoohi H (2023). Point-of-care ultrasound unveiling rotator cuff injuries in the emergency department: a case series. Cureus.

[14] Teefey SA, Rubin DA, Middleton WD, Hildebolt CF, Leibold RA, Yamaguchi K (2004). Detection and quantification of rotator cuff tears. Comparison of ultrasonographic, magnetic resonance imaging, and arthroscopic findings in seventy-one consecutive cases. J Bone Joint Surg Am.

[15] McGuire LB, Quinton AE, Cossetto DJ, Hanchard TJ, Spurway JF (2023). Diagnostic sensitivity of ultrasound of the supraspinatus tendon when compared to magnetic resonance imaging prior to arthroscopy: a retrospective study. Sonography.

[16] Farooqi AS, Lee A, Novikov D, Kelly AM, Li X, Kelly JD 4th, Parisien RL (2021). Diagnostic accuracy of ultrasonography for rotator cuff tears: a systematic review and meta-analysis. Orthop J Sports Med.

[17] Okoroha KR, Fidai MS, Tramer JS, Davis KD, Kolowich PA (2019). Diagnostic accuracy of ultrasound for rotator cuff tears. Ultrasonography.

